# Determinants of HIV testing uptake among adolescent girls and young women in mainland Tanzania: A stratified analysis of the 2016/17 and 2022/2023 national surveys

**DOI:** 10.1371/journal.pone.0343753

**Published:** 2026-07-08

**Authors:** Deogratius W. Kinoko, Anthony C. Kavindi, Paschal Yuda, Jovin R. Tibenderana, Ahmed Y. Nyaki, Sia E. Msuya, Michael J. Mahande

**Affiliations:** 1 Department of Epidemiology and Biostatistics, School of Public Health, KCMC University, Moshi, Tanzania; 2 Department of Public Health, St. Francis University College of Health and Allied Sciences, Ifakara, Tanzania; University of Health and Allied Sciences, GHANA

## Abstract

**Background:**

Adolescent girls and young women (AGYW) are disproportionately vulnerable to HIV. Despite expanded HIV testing services (HTS), the majority of AGYW remain unaware of their HIV status. This study aimed to assess determinants of HIV testing uptake among AGYW in mainland Tanzania before and after stratifying by age group (15–19 and 20–24 years) using data from two national surveys conducted over time.

**Methods:**

The data from the Tanzania HIV Impact Surveys (2016/17 and 2022/23), collected using multi-stage stratified sampling, were analyzed on 12,714 adolescent girls and young women. Data analysis was performed using STATA version 17. Weighted Modified Poisson regression models with robust standard error were fitted to identify factors associated with HIV testing uptake before and after stratifying by age group (15–19 and 20–24 years). Results were presented as adjusted prevalence ratio (APR) with a 95% confidence interval.

**Results:**

HIV testing uptake among adolescents remained stable at 40% in both the 2016/17 and 2022/23 surveys, while it increased among young women from 86% to 90% over the same period. Several factors were consistently associated with a higher prevalence of HIV testing uptake in both groups. Being in a union was more strongly associated with testing among adolescents (aPR = 1.52, 95% CI: 1.38–1.68) compared with young adults (aPR = 1.12, 95% CI: 1.07–1.17). Similarly, having secondary or higher education was associated with increased testing among adolescents (aPR = 1.22, 95% CI: 1.04–1.43) and young adults (aPR = 1.15, 95% CI: 1.06–1.25). A history of sexually transmitted infections (STIs) was also strongly associated with HIV testing uptake, particularly among adolescents (aPR = 1.53, 95% CI: 1.41–1.64), compared with young adults (aPR = 1.12, 95% CI: 1.09–1.15).

**Conclusions:**

HIV testing uptake among AGYW in Tanzania has improved over time, with significant disparities between adolescents and young women. These findings highlight the need for age-specific strategies, intensifying adolescent-focused interventions while sustaining efforts among young women and reinforcing integrated reproductive health and HIV services.

## Background

HIV/AIDS remains a major global public health concern, with Sub-Saharan Africa bearing the greatest burden of the global HIV epidemic [1]. In 2023, approximately 1.9 million AGYW aged 15–24 years were living with HIV worldwide, and most new infections in this group occurred in SSA [1]. In Tanzania, AGYW remain at a higher risk of HIV infection than males of the same age, highlighting the importance of improving HIV testing and prevention efforts among this vulnerable population [2].

Although Tanzania has made substantial progress in expanding HIV testing services, the persistent gap in HIV status awareness among AGYW underscores important considerations for the literature on national HIV testing policies and their effectiveness. National HIV testing strategies, led by the Ministry of Health Tanzania, and guided by global frameworks such as those from UNAIDS, emphasize universal access to testing through facility-based, community-based, and targeted approaches [3,4]. Another important policy dimension is the integration of HIV testing into broader health services, including STI management, family planning, and outpatient care [3]. Literature shows that such integration improves testing opportunities [5]. However, evidence from the literature suggests that policy availability does not necessarily translate into equitable uptake, particularly among adolescents [6]. For instance, according to THIS 2022/23, approximately 40.5% of HIV-positive AGYW are still unaware of their status in Tanzania, falling short of the first UNAIDS’ 95–95–95 targets [1,2]. Previous studies have revealed that approximately 44–66% of new HIV infections are from HIV-infected persons who are unaware of their HIV status [7].

Increasing the uptake of HIV testing and counselling would decrease the number of undiagnosed people [8]. Furthermore, increased HIV testing would work to increase awareness and truthful knowledge about HIV transmission, which is suitable for reducing HIV stigma and discrimination, known to adversely impact the uptake of HIV testing [8,9]. In addition, through HIV testing, people would have the opportunity to interact and discuss issues of HIV vulnerability, resilience, and transmission with trained health care providers in health care service spaces [9]. Using the factual knowledge acquired through such interactions, those testing negative for HIV can modify their lifestyles and practice safer sex as a resilience strategy [9].

Despite ongoing initiatives like Provider-Initiated HIV Testing and Counselling(PITC), Client-Initiated HIV Testing and Counselling (CITC), HIV self-testing(HIVST), and Determined, Resilient, Empowered, AIDS-free, and safe DREAMS [10,11]. Barriers such as stigma, limited availability of youth-friendly services, and age-related legal constraints continue to hinder testing uptake, particularly among adolescents aged 15–19 years [8,12]. For example, the UN Population Fund (UNFPA) recommends that access to information on Sexual reproductive health (SRH) and services be made available to people from the age of 10 years, and that the age of consent for HIV testing and counselling before and after testing without parental consent be lowered to 12 years. This approach has already been adopted in several countries in eastern and southern Africa, including Eswatini, South Africa, and Uganda [4]. However, in some countries, including Tanzania, the age of consent for HIV testing and counselling is set at 17 years and above [3]. This restriction may limit adolescents’ autonomy in accessing SRH services. Reduced autonomy in seeking SRH services may increase the likelihood of engaging in risky sexual behaviors, which heightens their risk for acquiring HIV infection [4].

A national survey conducted in Uganda reported ever testing among adolescent girls declined from 53.8% in 2016/17 to 48.7% in 2020/21, while among young women increased from 91.9% to 93.0% respectively [13]. Contrary to the national survey conducted in Tanzania that indicated ever tested for HIV among adolescent girls rose from 34.4% in 2011/12 and 38.2% in 2022/23, while among young women rose from78.4% to 89.3% respectively [2].

According to the study done in Zimbabwe, Malawi, and Tanzania, factors associated with higher HIV testing among AGYW included being aged 20–24years, living in an urban area, being married, having primary, secondary, or higher education, and having a history of pregnancy [14–17].

Previous studies have shown that age is a significant predictor of HIV testing uptake, with older adolescents and young women generally more likely to test than younger adolescents [16,18]. Additionally, key determinants of testing, such as educational attainment, marital status, health facility utilization, and antenatal care attendance, differ substantially between adolescents (15–19 years) and young women (20–24 years). However, many studies have analyzed these groups together, potentially masking important age-specific differences in testing behavior and associated factors. Stratified analysis by age group is therefore necessary to better understand these variations and to generate evidence that can inform age-appropriate HIV testing interventions.

Our study aimed to assess determinants of HIV testing uptake among AGYW in mainland Tanzania using data from two national surveys (THIS 2016/17and THIS 2022/23), stratified by age group, to identify disparities and inform targeted strategies.

## Conceptual framework

### Conceptual framework for analysis of factors associated with HIV testing uptake among AGYW in mainland Tanzania

**Fig 1:**
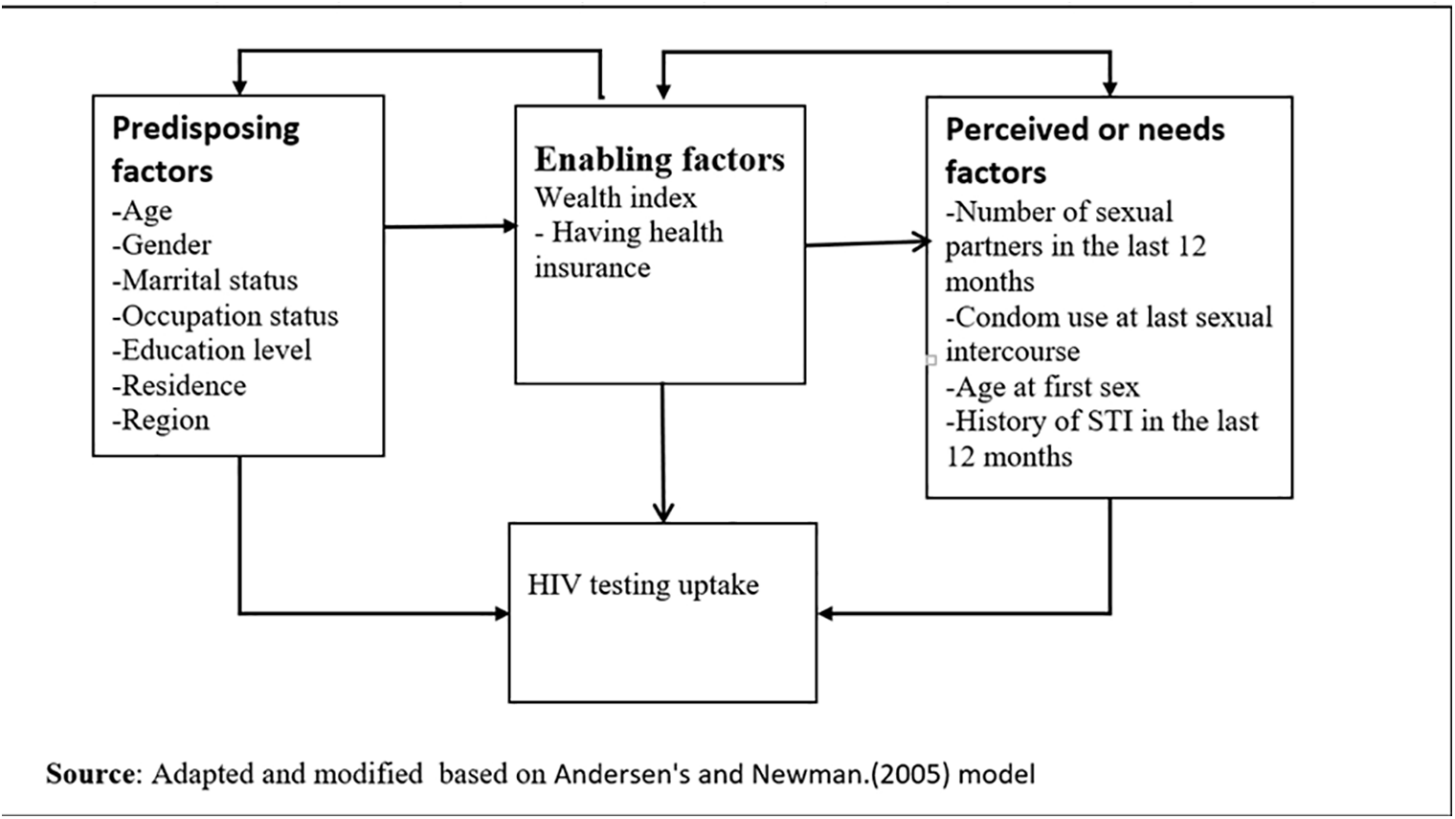
Conceptual framework for analysis of factors associated with HIV testing uptake among AGYW.

We adopted Andersen’s Behavioral Model of Health Services Utilization to conceptualize the relationship between individual and contextual factors and HIV testing uptake among adolescent girls and young women (AGYW) [19]. This model has been widely applied in studies examining healthcare utilization, including HIV-related behaviors [5,9,20]. The framework considers health service use as a function of three domains: predisposing factors, enabling factors, and need factors [19]. In the context of HIV testing, the model assumes that an individual’s likelihood of utilizing HIV testing services is shaped by their socio-demographic background and beliefs (predisposing factors), the availability and accessibility of resources that facilitate service use (enabling factors), and their perceived or evaluated health needs (need factors) [19]. These domains interact to influence whether AGYW decide to seek and obtain HIV testing services (Fig 1).

**Predisposing factors** refer to characteristics that exist before illness and influence an individual’s propensity to use health services [5,9]. These are background characteristics that shape an individual’s likelihood of using health services even before a need arises. They influence beliefs, knowledge, and attitudes toward HIV testing. For example, the younger women cohort (20–24 years) tends to be more conscious about meeting their health care needs, and that of their dependents, may be more inclined to use health care services, including testing for HIV, relative to others [9]. For instance, factors such as marital status, gender, province of residence, educational attainment, and employment status are known to influence the utilization of health care services. For example, among married individuals, the perception of a reduced risk of HIV infection may discourage regular use of HIV testing services compared to their unmarried counterparts [5,9].

**Enabling factors** represent the logistical and resource-related conditions that either facilitate or hinder access to HIV testing services [5]. The enabling variables include: Household wealth index, Health insurance coverage, and availability of HIV testing services in the community. These factors determine whether AGYW who are predisposed to test are practically able to access testing services. For instance, people who are from households with a higher wealth index have increased HIV testing compared to their counterparts [5,9].

**Perceived/needs factors** reflect an individual’s perception that they are very susceptible or at increased risk of mortality from a given sickness or illness; they tend to perceive a heightened need for the use of health care services to address the health challenges [5,9]. In relation to HIV testing, these include: Number of sexual partners, History of sexually transmitted infection (STI) symptoms, and Sexual activity status. These variables capture both perceived risk of HIV infection and clinical indications that may prompt HIV testing.

Within this framework, HIV testing uptake among AGYW is viewed as a health-seeking behavior influenced by: Predisposition, Ability to access services, and Perceived risk or need. Thus, the Andersen model provides a structured approach for examining how socio-demographic, economic, and behavioral factors jointly shape HIV testing utilization, making it particularly suitable for analyzing population-based survey data such as the Tanzania HIV Impact Survey.

## Materials and methods

### Study design and study area

This study used a cross-sectional study design, analyzing data from the Tanzania HIV impact survey conducted between 2016/17 and 2022/23, and the dataset was accessed from the Population-based HIV Impact Assessment on 23/04/2025. Tanzania is the largest country in East Africa, spanning approximately 940,000 square kilometers, including about 60,000 square kilometers of inland water. As of 2022, the estimated AGYW population in mainland Tanzania was 5,988,919 [21].

Tanzania HIV/impact surveys implemented by the National Bureau of Statistics (NBS), the Office of the Chief Government Statistician (OCGS) Zanzibar, the National AIDS, STIs and Hepatitis Control Programme (NASHCoP), and the Zanzibar Integrated HIV, Hepatitis, Tuberculosis and Leprosy Program (ZIHHTLP), with technical assistance from the US Centers for Disease Control and Prevention (CDC) and ICAP at Columbia University. The surveys are funded by the US President’s Emergency Plan for AIDS Relief (PEPFAR).

### Sampling design

The Tanzania HIV Impact Survey (THIS) employed a two-stage stratified cluster sampling design to obtain nationally representative estimates [2]. In the first stage, enumeration areas (EAs) were selected with probability proportional to size within strata defined by region and urban–rural residence. In the second stage, households were systematically selected from updated household listings within each selected EA. Sampling weights were applied to account for unequal selection probabilities at each stage of sampling and for household and individual non-response. Weights were calculated as the inverse of the probability of selection of each household and individual. These weights were subsequently adjusted for non-response and calibrated to match national population distributions by age, sex, and region using post-stratification. All analyses incorporated sampling weights, clustering, and stratification to produce nationally representative estimates and correct standard errors.

### Study population and sample size

**Fig 2:**
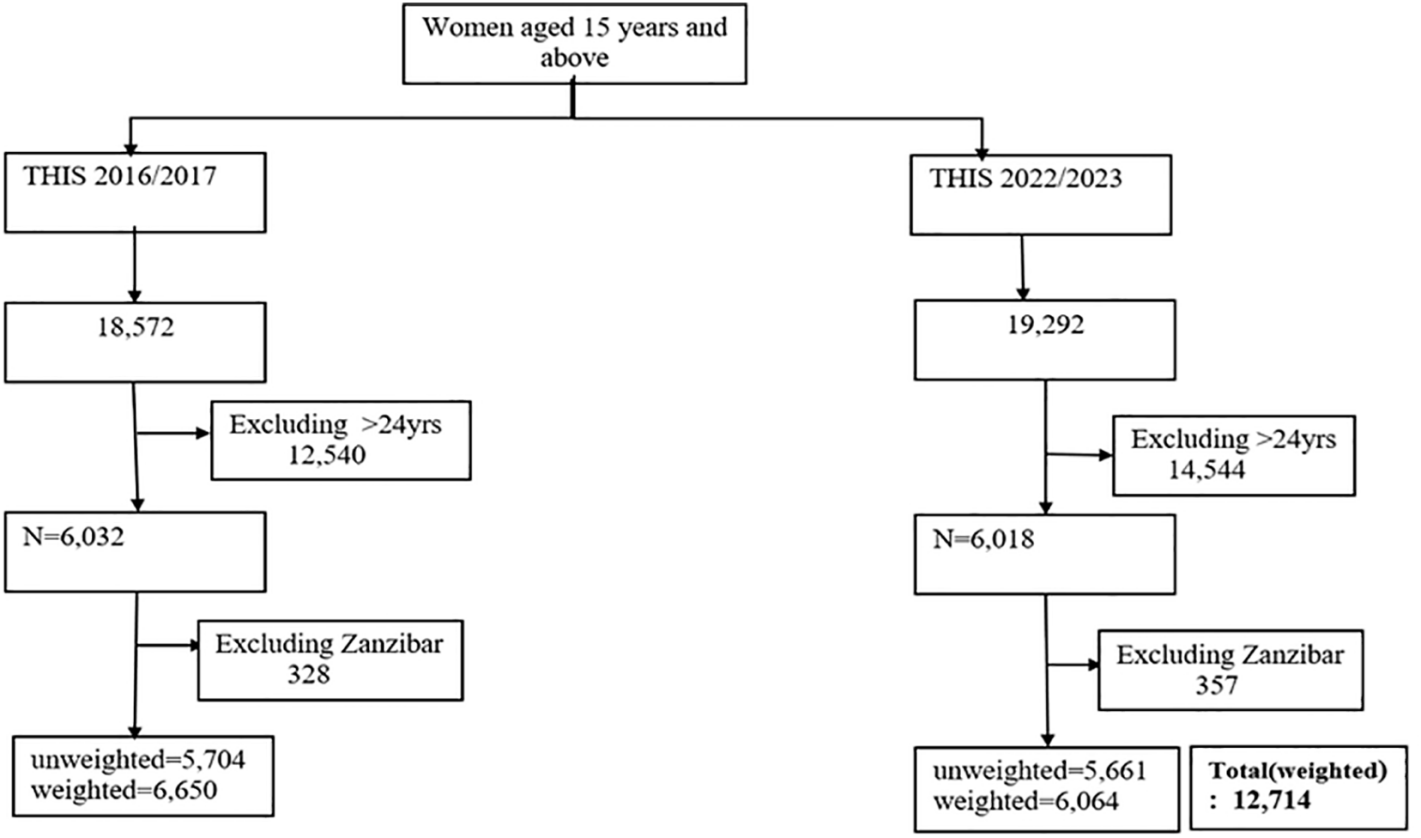
Flow chart for selection of study participants (Adolescent Girls and Young Women).

All AGYW from mainland Tanzania interviewed in the Tanzania HIV Impact Survey between 2016/17 and 2022/23 were included in the study. The number of AGYW [15–24] included in the study in the years 2016/17 and 2022/23, after applying weighting, was 6,650 and 6,064, respectively. The total weighted sample size of all AGYW included in the study was 12,714 (Fig 2)

### Study variables

#### Outcome variable.

The outcome of interest in this study was whether the AGYW had ever tested for HIV and received results, a binary variable if the AGYW reported “Yes” if ever been tested for HIV and received results, and “No” if not ever tested for HIV, coded as 1 “Yes” and 0 “No”.

#### Independent variables.

Independent variables were classified into Predisposing factors, enabling factors, and perceived or needs factors. The predisposing factors includes: age of respondents (0 = 15–19 and 1 = 20–24years), place of residence (0 = Rural, and 1 = Urban), administrative zone (1 = Central, 2 = Lake, 3 = Northen, 4 = Eastern, 5 = Southern West Highland, 6 = Southern Highland, 7 = Southern, 8 = Western), marital status(0 = Never in union, 1 = Currently in union, 2 = Cohabiting and 3 = formerly in union), occupation status(0 = Not employed, 1 = Employed), education level(0 = No education, 1 = Primary education and 2 = secondary education and above), exposure to TV/radio(0 = No and 1 = Yes). Enabling factors include: having had health insurance (0 = No, and 1 = Yes), Wealth index (0 = poor,1 = middle, and 2 = Rich). Perceived or needs factor includes: age at first sex (0=<15, 1 = 15+), multiple sex partners in the last 12 months (0 = No partner, 1 = one, 2 = two and above), having had STI in the last 12 months (0 = No, 1 = Yes), condom use in the last sex (0 = No and 1 = Yes). HIV results from the biomarker test (0 = Negative, 1 = Positive).

Zones rather than administrative regions were used in order to have consistency across the surveys. All regions belong to the same zones, and the geographical coverage of the zones has remained consistent throughout the surveys. Composition of the administrative regions in their respective zones is as follows: Eastern (Morogoro, Pwani, Dar es Salaam); Northern (Kilimanjaro, Tanga, Arusha); Lake (Mwanza, Geita, Mara, Simiyu, Shinyanga, Kagera); Central (Dodoma, Manyara, Singida); Western (Kigoma, Tabora); South West Highlands (Katavi, Rukwa, Mbeya, Songwe); Southern Highlands (Iringa, Njombe, Ruvuma); and Southern (Lindi, Mtwara)(S1 Table).

### Data management and analysis

Data cleaning and analysis were performed using STATA version 17.0. The Variables were categorized or recategorized based on previous literature and plausibility [16,22,23]. Unique ID variables merged the adult individual and biomarker datasets, and then appended across survey years to form a pooled dataset for analysis. The data analysis accounted for the complex survey design by incorporating survey weights, primary sampling units (clusters), and strata using the svyset command applied to the defined subpopulation.

Descriptive analyses are shown in frequencies and percentages for categorical variables and continuous variables using the mean with respective standard deviation to describe the AGYW characteristics. The Cochran-Armitage test for trend was used to identify significant changes in HIV testing uptake across survey years.

To determine factors associated with HIV testing uptake among AGYW, the classical logistic regression was considered. However, this approach was not used because the prevalence of HIV testing among AGYW was greater than 10% even after stratification, due to the limitation of logistic regression of overestimating odds ratios and 95%CI for outcomes with prevalence greater than 10%. Therefore, log binomial regression was then considered, but our analysis failed to converge. Finally, a modified Poisson regression model was used [24]. Its functional form is in equation (i) below.


$$\begin{array}{c} og\;(\pi i)=\beta \text{0}+\beta_{\text{1}}X_{\text{1}}i+\cdots +\beta kXk\text{ἱ} \end{array}$$ (i)

where π𝑖 is the probability of experiencing the outcome of interest for subject 𝑖

β’s is the mean of the 𝑖^th^ subject and approximates relative ratios as exp(β).

The analysis was stratified into age groups to reflect critical differences in legal, social, developmental, and contextual factors that may influence HIV testing behaviors among adolescents and young people in mainland Tanzania. The age groups were categorized as 15–19 and 20–24 years to capture meaningful differences related to legal autonomy, maturity, and social expectations.

In Tanzania, adolescents under the age of 18 are typically required to obtain parental or guardian consent to access HIV testing services [3]. This legal barrier can significantly impact access and uptake. Furthermore, developmental differences and varying levels of autonomy and risk perception between younger and older adolescents necessitate separate analysis. Combining these age groups could obscure important patterns and hinder the identification of age-specific predictors of HIV testing. Therefore, separate analyses were conducted to ensure meaningful interpretation and policy-relevant insights.

The pooled modified Poisson regression model estimated the Prevalence ratios (PR) with their corresponding 95%CI in one survey phase (2016/17–2022/23). Bivariable modified Poisson regression analyses with robust standard errors were conducted to assess the association between each independent variable and the outcome. Variables with p < 0.20 were considered for inclusion in the multivariable model to avoid excluding potential confounders at an early stage. A multivariable modified Poisson regression model was then fitted to estimate adjusted prevalence ratios (aPRs). Multicollinearity was assessed using the Variance Inflation Factor (VIF). Variables with VIF ≥ 10 were considered highly collinear and were excluded or retained based on epidemiological relevance. Known confounders such as zone and residence were retained in the model regardless of statistical significance. Variables were retained in the final model based on statistical significance, epidemiological plausibility, and their impact on the effect estimates of key exposures, with a change-in-estimate threshold of 10% used to identify confounders.

To assess the robustness of the findings, sensitivity analyses were conducted by fitting alternative models, including survey-weighted logistic regression. Additionally, age-stratified analyses were performed to examine potential differences between adolescents (15–19 years) and young women (20–24 years). Consistency in the direction and significance of associations across models was used to evaluate robustness. Sensitivity analyses were conducted to assess the robustness of the findings. These included comparing results from modified Poisson regression with logistic regression. Additional analyses excluding potential mediators and stratified by age group were also performed.

## Ethical clearance

Ethical approval to conduct the study was sought from the KCMC University Research and Ethics Review Committee (KURERC) with clearance number PG 180/2024, dated 7^th^ October, 2024. Permission to access the datasets was granted by the Tanzania National Data Archive (TNADA) and the PHIA project website following submission of a project abstract on 23^rd^ April, 2025. The study used anonymized secondary data; thus, informed consent was not required. Confidentiality was preserved by excluding all personal identifiers from the analysis and reporting.

## Results

### Background characteristics of the study participants

A total of 12,714 participants were analyzed across the two surveys, with a mean (SD) age of 19.4(±2.8)**.** Across the two surveys (2016/17 and 2022/23), the majority of participants were aged 15–19 years, although their proportion decreased over time from 54.2% in 2016/17 to 51.4% in 2022/23. Additionally, the majority resided in rural areas, and their proportion remained constant 59.3% in the years 2016/17 and 2022/23. The majority of AGYW were never married, with the proportion remaining relatively consistent at approximately 56% throughout all survey years. Regarding education, most AGYW had attained primary education, although this proportion declined over time from 55% in 2016/17–48% in 2022/23. The majority of AGYW reported having one sexual partner, with proportions increasing from 51.7% in 2016/17 to 63.9% in 2022/23. Additionally, the majority of AGYW reported having had no sexually transmitted infection (STI) in the 12 months before the survey, with the proportions dropping from 87.5% in 2016/17 to 81.3% in 2022/23 (S2 Table).

### Trends of HIV testing uptake among adolescent girls and young women

**Fig 3:**
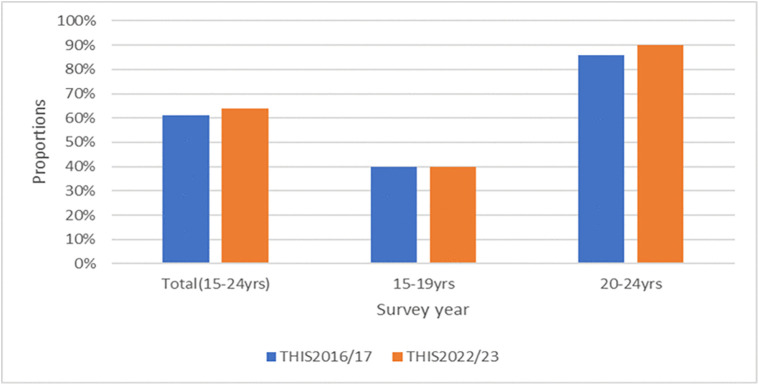
Trends in ever tested for HIV and received results among AGYW in mainland Tanzania between THIS2016/17, and 2022/23.

Trends in HIV testing among AGYW (15–24years) who had ever been tested and received results, the proportion increased from 61% in 2016/17 and 64% in 2022/23**.** When stratified by age, HIV testing uptake among adolescent girls aged 15–19 years remained 40% in both 2016/17 and 2022/23 surveys. In contrast, among young women aged 20–24 years, HIV testing increased from 86% to 90%, respectively (Fig 3).

### Distribution of HIV testing uptake according to selected characteristics

HIV testing uptake differed markedly between adolescents (15–19 years) and young women (20–25 years) across both survey periods (2016/17 and 2022/23). Overall, testing coverage was consistently substantially higher among young women, with proportions exceeding 85% in most subgroups, compared to considerably lower levels among adolescents, which ranged between approximately 28% and 54% (S3 Table).

Between 2016/17 and 2022/23, young women demonstrated notable improvements in HIV testing uptake across most characteristics. For instance, testing among those currently in union rose from 91.9% to 95.8% (p < 0.001). In contrast, adolescents showed minimal or inconsistent changes over time, with improvement observed among adolescents currently in union from 72.5% to 84.8% (p < 0.001)

HIV testing uptake varied across behavioral and health-related characteristics among adolescents (15–19 years) and young women (20–24/25 years), with consistently higher coverage among young women than among adolescents in both survey periods. For instance, among adolescents, HIV testing uptake was substantially higher among those who did not use a condom at last sex from 63.5% to 73.8% (P < 0.001). A similar pattern was observed among young women, where testing uptake was higher among those who did not use condoms from 90.3% to 93.6%(P < 0.001). Testing uptake was strongly associated with recent STI experience. Among adolescents, those reporting an STI in the past 12 months showed a substantial increase in testing uptake from 55.5% to 82.3% (p < 0.001). Among young women, testing uptake was consistently higher among those with a recent STI from 87.7% to 97.7% (p < 0.001).

### Proportion of HIV testing uptake among AGYW by regions

**Fig 4:**
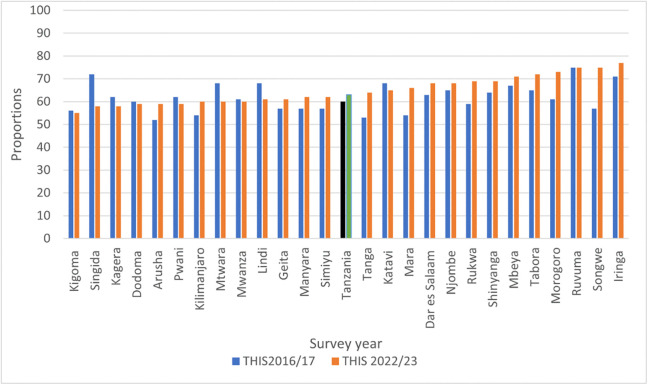
Trends in ever tested for HIV and received results among AGYW in mainland Tanzania by regions 2016/17, and 2022/23.

Between 2016/17 and 2022/23, half of the regions in mainland Tanzania recorded a significant increase in HIV testing among AGYW beyond the national average (63%). These regions are Tanga, Katavi, Mara, Dar es-Salaam, Njombe, Rukwa, Shinyanga, Mbeya, Tabora, Morogoro, Ruvuma, Songwe, and Iringa. These regional variations highlight both progress and gaps in HIV testing efforts, underscoring the need for targeted interventions in areas where proportions have declined (Fig 4)**.**

### Factors Associated with HIV Testing Uptake among AGYW in Mainland Tanzania (2016/17–2022/23)

S4 Table presents the results from pooled multivariable modified Poisson regression models assessing factors associated with HIV testing uptake among AGYW in mainland Tanzania. HIV testing prevalence was 31% higher among AGYW aged 20–24 years compared to those aged 15–19 (APR: 1.31, 95% CI: 1.26–1.37, p < 0.001), indicating age as a strong predictor of testing uptake. However, a 42% reduction in the adjusted prevalence ratio suggests that the effect of age is partially confounded by other variables such as marital status and sexual activity.

Being in a union was consistently associated with higher HIV testing uptake. AGYW who were currently married, cohabiting, or formerly in union had 29%, 25%, and 20% higher prevalence of testing, respectively, compared to those never in union. Yet, after adjusting for confounders, the strength of these associations decreased by 39–49%, indicating that marital status likely acts as a proxy for other underlying factors, particularly age and sexual exposure.

Education also played a significant role. AGYW with at least secondary education had a 16% higher prevalence of HIV testing compared to those with no formal education (APR: 1.16, 95% CI: 1.07–1.26, p < 0.001), reflecting the positive impact of educational attainment on health-seeking behavior. Recent STI history emerged as a strong independent predictor. AGYW who reported having had an STI in the past 12 months had a 22% higher likelihood of undergoing HIV testing (APR: 1.22, 95% CI: 1.18–1.26, p < 0.001), underscoring how perceived risk increases demand for testing.

Sexual behavior also influenced testing. AGYW with one sexual partner had 30% higher HIV testing prevalence than those who had never had a partner (APR: 1.30, 95% CI: 1.00–1.90, p = 0.030). However, this association weakened by 44% after adjustment, suggesting it was likely confounded by other risk indicators such as age, marital status, and STI experience.

### Factors associated with HIV testing among AGYW in mainland Tanzania stratified by age using data from THIS 2016/17 and 2022/23

When examining factors associated with HIV testing uptake among AGYW in mainland Tanzania stratified by age groups (adolescents aged 15–19 and young women aged 20–24) using data from the 2016/17 and 2022/23 THIS surveys, similar predictors were identified across both groups. These included being in a union (currently married, cohabiting, or formerly in a union), attaining secondary education or higher, and having had a sexually transmitted infection (STI) within the past year (S5 Table). The consistency of these associations suggests common individual-level drivers of HIV testing across the adolescent and young adult age spectrum.

However, after adjusting for potential confounders, the association between marital status and HIV testing uptake among adolescents aged 15–19 was notably attenuated. Prevalence ratios dropped by 43% for those currently in union, 42% for those cohabiting, and 44% for those formerly in union. This suggests that marital status in this age group may not independently influence HIV testing, but rather acts as a proxy for other factors such as age, sexual debut, or STI risk, each of which more directly motivates testing.

In contrast, the corresponding reductions among young women aged 20–24 were modest (7% to 9%), indicating that marital status remains a more stable, independent predictor of HIV testing in this older cohort. This could reflect greater autonomy, increased exposure to health services, or higher perceived HIV risk post-marriage.

Our findings underscore the importance of considering age-specific dynamics when designing HIV testing interventions. For adolescents, strategies may need to go beyond marital status and directly address sexual health education, STI prevention, and accessibility of youth-friendly HIV testing services.

### Sensitivity analysis

Sensitivity analysis using survey-weighted logistic regression yielded results consistent in direction and statistical significance with those obtained from the modified Poisson regression model. Key predictors such as age, marital status, education level, condom use, and history of vaginal discharge remained significantly associated with HIV testing uptake. However, effect sizes were larger in the logistic model, reflecting the high prevalence of the outcome. Therefore, the use of modified Poisson regression to estimate prevalence ratios was deemed more appropriate (S6 Table). Age-stratified analyses further showed that the direction of associations was generally consistent among adolescents (15–19 years) and young women (20–24 years), with slightly stronger effects observed among young women, particularly for marital status and education level.

## Discussion

The findings from this study indicate that the overall proportion of HIV testing uptake among AGYW increased from 61% in 2016/17–64% in 2022/23. This incremental progress aligns with broader regional trends in Sub-Saharan Africa, where expansion of HIV testing services has contributed to gradual improvements in coverage, though often insufficient to meet the UNAIDS 95-95-95 targets. The observed increase likely reflects the scale-up of diversified testing strategies, including PITC, community-based, and HIV self -testing, as promoted in national HIV programs.

After stratified age, the substantial increase in testing coverage among women aged 20–24 years (from 86% to 90%) is likely driven by their higher engagement with maternal and reproductive health services, particularly antenatal care (ANC). In Tanzania, national HIV testing guidelines developed by the National AIDS Control Programme Tanzania under the Ministry of Health Tanzania strongly emphasize routine provider-initiated HIV testing during ANC visits [3]. This policy has been instrumental in normalizing HIV testing and integrating it into routine care, thereby increasing coverage among women of reproductive age. Similar successes have been reported in other settings where ANC-based HIV testing has served as a key entry point into HIV services [16,25]. This finding is largely consistent with the literature, where it is explained that younger adults and the middle-aged exercise greater autonomy over their health and that of their dependents. In contrast, the persistently low uptake among adolescents (15–19 years), which remained unchanged at 40%, highlights a critical gap in current HIV testing strategies. This stagnation suggests that existing approaches may not adequately address the unique barriers faced by adolescents. Structural and policy-related factors, particularly legal requirements for parental consent for HIV testing among individuals under 18 years, may restrict adolescents’ timely access to HIV testing services [3,4]. In contrast, evidence from eastern and southern Africa, including Lesotho, Eswatini, South Africa, and Uganda, where policies allow adolescents to independently consent to HIV testing from around age 12, suggests that more enabling legal environments can facilitate earlier and increased uptake of HIV testing among adolescents [4,15]. Beyond legal constraints, other barriers such as stigma, concerns about confidentiality, limited availability of adolescent-friendly services, and low perceived risk have been consistently reported in the literature as key deterrents to HIV testing among adolescents [26]. Studies across sub-Saharan Africa suggest that adolescents are less likely to access facility-based services due to fear of judgment and lack of privacy, underscoring the need for differentiated service delivery models.

This study revealed that HIV testing uptake changes with women aged 20–24 years having a higher prevalence of getting tested for HIV than women aged 15–19 years. This finding is consistent with previous studies [6,14–17]. This could be explained by the fact that women aged 20–24 years of age are more likely to be sexually active, married, cohabiting, or pregnant, which increases their exposure to routine provider-initiated HIV testing through antenatal and reproductive health services. Additionally, the observed lower testing uptake among adolescents aged 15–19 years may be related to structural and social barriers, including dependence on parental consent, fear of stigma, and lower perceived HIV risk—factors that have been reported in studies among adolescents in sub-Saharan Africa. Eventually, these structural, behavioral, and service-related factors collectively drive higher HIV testing uptake among these women.

Marital status was positively associated with HIV testing, with higher uptake observed among women who were currently in union, cohabiting, or formerly in union. These findings are aligned with previous studies in Tanzania and elsewhere [6,9,22,25,27]. The following could be reasons for these findings: partnered women have increased contact with health services through antenatal care, family planning, and couple-based interventions where testing is routinely recommended. Being in or having been in a partnership heightens perceived HIV risk related to partner fidelity and future childbearing, motivating acceptance of testing. Furthermore, healthcare providers may prioritize testing among partnered women to support prevention of mother-to-child transmission and partner notification, while social support from partners can reduce fear, stigma, and anxiety

The history of sexually transmitted infections (STIs) was also strongly associated with HIV testing, likely reflecting the integration of HIV testing into STI diagnosis and treatment services. Similar associations have been reported in Senegal and Haiti, where individuals with STI symptoms were more likely to seek or be referred for HIV testing [28]. However, earlier studies in Tanzania and Zimbabwe reported no such association [14,16]. The observed association between a history of sexually transmitted infections (STIs) and HIV testing uptake can be interpreted within the framework of national HIV policies in Tanzania. Specifically, the implementation of provider-initiated testing and counseling (PITC) under the Ministry of Health Tanzania mandates that HIV testing be routinely offered to individuals presenting at health facilities, particularly those with conditions indicative of elevated HIV risk, such as STIs(3). This policy framework ensures that individuals seeking STI diagnosis or treatment are systematically screened for HIV, thereby increasing testing uptake among this group.

Furthermore, the integration of HIV testing into routine clinical services is reinforced through the National AIDS Control Programme (NACP), which promotes comprehensive and integrated service delivery across HIV, STI, tuberculosis, and reproductive health services. Under this approach, STI services serve as key entry points for HIV testing, counseling, and linkage to care [3]. The substantial increase in HIV testing uptake among individuals reporting recent STIs in 2022/23 likely reflects improved implementation and scale-up of these integrated service delivery models, particularly at primary healthcare levels.

Education level emerged as another important determinant, with AGYW who had completed secondary education or higher being significantly more likely to have ever tested for HIV. This association is consistent with findings from multiple countries, including Zambia, Burundi, and South Africa [5,29]. Increased educational attainment may enhance awareness of HIV transmission, reduce stigma, and empower young women to access testing services. This finding aligns with the objectives of the Ministry of Health and the Ministry of Education, Science, and Technology in Tanzania, and the United Nations Population Fund (UNFPA), which promote the integration of comprehensive sexuality education (CSE) within school curricula. National frameworks such as the School Health Programme and adolescent sexual and reproductive health (ASRH) strategies emphasize equipping young people with knowledge and life skills to make informed health decisions, including HIV testing [30,31].

## Strengths and limitations

This study is the first in Tanzania to comprehensively examine determinants of HIV testing among AGYW by age strata, using robust nationally representative survey data spanning over a decade. The large sample sizes provided sufficient power for subgroup analysis.

However, the study is not without limitations. The use of secondary data introduces potential issues related to missing or unmeasured variables. Self-reported HIV testing history may be subject to recall or social desirability bias, particularly among adolescents. Additionally, certain important contextual and behavioral variables (e.g., stigma, peer influence, knowing a place for HIV testing, alcohol use, and sexual violence) were not captured in the datasets consistently, limiting the interpretation of some observed associations.

## Recommendations

Promoting couple HIV testing and counselling (CHTC) among AGYW in unions or cohabiting relationships can facilitate mutual disclosure, reduce stigma, and encourage shared responsibility in HIV prevention and care.

Integration of HIV testing with sexual and reproductive health (SRH) services should be strengthened. Programs should continue to promote routine HIV testing within STI care while expanding access through youth-friendly health service platforms to improve uptake among adolescent girls and young women.

School-based sexual and reproductive health (SRH) education should be strengthened, with efforts aimed at improving its quality, coverage, and effectiveness. In addition, policy reforms regarding adolescent consent for HIV testing and related SRH services are needed to reduce barriers to service uptake. The Tanzania Education and Training Policy (ETP) 2014 Edition of 2023 outlines several key areas, including the integration of health education in primary, secondary, and higher levels, to overcome health challenges, including HIV prevention [30].

Further qualitative studies are needed to explore social, cultural, and psychological barriers to HIV testing among adolescents, particularly those aged 15–19 years.

## Conclusion

HIV testing uptake among AGYW in mainland Tanzania has improved between 2016/72 and 2022/2023, with notable gains among young women aged 20–24 years. However, progress among adolescents remains limited. Key factors consistently associated with a higher prevalence of HIV testing include being in a union, a history of STIs, and higher educational attainment. Targeted efforts are needed to address persistent barriers and expand access to adolescent-friendly HIV testing services.

## Supporting information

S1 Table Coding and categorization of independent variables. (DOCX)

S2 Table Background characteristics of the study participants (weighted) in mainland Tanzania (N = 12,714). (DOCX)

S3 Table Trends of ever tested for HIV and received results by selected characteristics of study participants (Chi-square test) in THIS2016/17, and THIS2022/23 (N = 12,714). (DOCX)

S4 Table Multivariable Poisson regression on factors associated with HIV testing among AGYW in mainland Tanzania using data from THIS 2016/17 and 2022/23 (N = 12,714). (DOCX)

S5 Table Multivariable modified Poisson regression on factors associated with HIV testing among AGYW in mainland Tanzania stratified by age using data from THIS 2016/17 and 2022/23 (N = 12,714). (DOCX)

S6 Table Multivariable logistic regression on factors associated with HIV testing among AGYW in mainland Tanzania using data from THIS 2016/17 and 2022/23 (N = 12,714). (DOCX)

S2 Data Analytical data for the study. (RAR)

## Abbreviations

AGYW: Adolescent Girl and Young Women
CHTC: Couple HIV Testing and Counselling
CITC: Client-Initiated HIV Testing and Counselling
DHS: Demographic Health Survey
HIV: Human Immune Deficiency Virus
HIVST: HIV Self Testing
HTS: HIV Testing Services
MoH: Ministry of Health
NASHCoP: National AIDS, STIs and Hepatitis Control Programme
PITC: Provider-Initiated HIV Testing and Counselling
STIs: Sexually Transmitted Infections
THIS: Tanzania HIV Impact Survey
UNAIDS: United Nations Programme on HIV/AIDS
VCTC: Voluntary Testing and Counselling Center
WHO: World Health Organization
