# Supplementary material for: Determinants of HIV testing uptake among adolescent girls and young women in mainland Tanzania: A stratified analysis of the 2016/17 and 2022/2023 national surveys
Source: PLoS One. 2026 Jul 8;21(7):e0343753. doi: 10.1371/journal.pone.0343753 (PMC13345389; doi:10.1371/journal.pone.0343753)
Supplement: S1 Table — (DOCX) [file pone.0343753.s001.docx]

**S1 Table: Coding and categorization of independent variables**

| S/N | Variable | Coding |
| --- | --- | --- |
|  | **Predisposing factors** |  |
| 1 | Age | 0=15-19 and 1=20-24years |
| 2 | place of residence | 0=Rural, and 1=Urban |
| 3 | zone | 1=Western, 2=Northern, 3=Central, 4=Southern Highlands, 5=Southern, 6=South West Highlands, 7=Lake, 8=Eastern, 9=Zanzibar |
| 4 | marital status | 1=Not married, and 2=married |
| 5 | Education level | 0=No education, 1=Primary education and 2=secondary education and above |
| 6 | Occupation | 0=Not employed and 1=Employed |
| 7 | Exposure to TV and Radio | 0=No and 1=Yes |
|  | **Enabling factors** |  |
| 8 | Wealth index | 0=poor,1=middle and 2=Rich |
| 9 | Health insurance | 0=No, and 1=Yes |
|  | **Needs factors** |  |
| 10 | Age at first sex | 0=<15, 1=15-19, and 2=20-24) |
| 11 | Had Multiple sex within the last 12 months | 0=No partner, 1=one, 2=two and above) |
| 12 | Last time you had sex, was a condom used | 0=No and 1=Yes |
| 13 | HIV test results from the biomarker test | 0=Negative and 1=Positive |
