# Supplementary material for: Determinants of HIV testing uptake among adolescent girls and young women in mainland Tanzania: A stratified analysis of the 2016/17 and 2022/2023 national surveys
Source: PLoS One. 2026 Jul 8;21(7):e0343753. doi: 10.1371/journal.pone.0343753 (PMC13345389; doi:10.1371/journal.pone.0343753)
Supplement: S2 Table — (DOCX) [file pone.0343753.s002.docx]

**S2 Table. Background characteristics of the study participants (weighted) in mainland Tanzania (N=12,714)**

| **Survey year** | | |
| --- | --- | --- |
| **Characteristics** | **2016/2017 (n=6,650)**  **n(%)** | **2022/2023 (n=6,064)**  **n(%)** |
| **Age(years)** |  |  |
| 15-19 | 3,603(54.2) | 3,115(51.4) |
| 20-24 | 3,047(45.8) | 2,949(48.6) |
| **Mean (SD)** | 19.4(2.8) | 19.6(2.8) |
| **Residence** |  |  |
| Rural | 3,943(59.3) | 3,600(59.4) |
| Urban | 2,707(40.7) | 2,464(40.6) |
| **Zone** |  |  |
| Central | 698(10.5) | 542(8.9) |
| Lake | 1,129(27.5) | 1,928(31.8) |
| Northern | 750(11.3) | 640(10.6) |
| Eastern | 1,309(19.7) | 1,234(20.4) |
| South West Highland | 813(12.2) | 594(9.8) |
| Southern Highland | 489(7.4) | 322(5.3) |
| Southern | 190(2.9) | 226(3.7) |
| Western | 571(8.6) | 577(9.5) |
| **Marital status** |  |  |
| Never in union | 3,705(55.7) | 3,383(55.8) |
| Currently in a union | 1,875(28.2) | 1,839(30.3) |
| Cohabiting | 706(10.6) | 531(8.8) |
| Formerly in union | 364(5.5) | 312(5.1) |
| **Occupation status** |  |  |
| Not employed | 4,952(74.5) | 4,323(71.3) |
| Employed | 1,699(25.5) | 1,741(28.7) |
| **Education Level** |  |  |
| No education | 551(8.3) | 466(7.7) |
| Primary education | 3,655(55) | 2,913(48) |
| Secondary/Higher | 2,445(36.7) | 2,685(44.3) |
| ᵃ-Sex debut 2016/17(n=4,677); ᵇ-Multiple sexual partners 2016/17(n=6,612);  ᶜ-Condom use 2011/12(n=2,516),2016/17(n=6,089),2022/23(n=5,804); ᵉ-Had STI in last 12months 2016/17(n=4,677), 2022/23(n=5,469) | | |

**S2 Table. (Continued)**

|  | **Survey year** | |
| --- | --- | --- |
| **Characteristics** | **2016/17(n=6650)**  **n(%)** | **2022/23(n=6064)**  **n(%)** |
| **Wealth index** |  |  |
| Poor | 2,417(36.3) | 2,472(40.8) |
| Middle | 1,389(20.9) | 1,229(20.3) |
| Rich | 2,845(42.8) | 2,363(38.9) |
| **Exposure to Radio/TV** |  |  |
| No | 2,417(36.3) | 1,842(30.4) |
| Yes | 4,234(63.7) | 4,222(69.6) |
| **Had health insurance** |  |  |
| No | 5,971(89.8) | 4,817(79.4) |
| Yes | 680(10.2) | 1,247(20.6) |
| **Age at first sex ᵃ** |  |  |
| <15 | 615(13.2) | 351(8.7) |
| 15+ | 4,062(86.8) | 3,665(91.3) |
| \| **Multiple sexual partners ᵇ** \| \| --- \| |  |  |
| No partner | 583(8.8) | 298(5.0) |
| One | 3,421(51.7) | 3,642(60.7) |
| Two and above | 2,608(39.5) | 2,058(34.3) |
| \| **Condom use in last sex ᶜ** \| \| --- \| |  |  |
| No | 3,197(52.5) | 3,327(57.3) |
| Yes | 2892(47.5) | 2,477(42.7) |
| **Had STI in the last 12monthsᵉ** |  |  |
| No | 4,091(87.5) | 4,447(81.3) |
| Yes | 586(12.5) | 1,022(18.7) |
| \| **HIV results from**  **the biomarker test.** \| \| --- \| |  |  |
| Positive | 141(2.1) | 78(1.3) |
| Negative | 6,509(97.9) | 5,986(98.7) |
| ᵃ-Sex debut 2016/17(n=4,677); ᵇ-Multiple sexual partners 2016/17(n=6,612);  ᶜ-Condom use 2011/12(n=2,516),2016/17(n=6,089),2022/23(n=5,804); ᵉ-Had STI in last 12months 2016/17(n=4,677), 2022/23(n=5,469) | | |
