# Supplementary material for: Determinants of HIV testing uptake among adolescent girls and young women in mainland Tanzania: A stratified analysis of the 2016/17 and 2022/2023 national surveys
Source: PLoS One. 2026 Jul 8;21(7):e0343753. doi: 10.1371/journal.pone.0343753 (PMC13345389; doi:10.1371/journal.pone.0343753)
Supplement: S3 Table — (DOCX) [file pone.0343753.s003.docx]

**S3 Table. Trends of ever tested for HIV and received results by selected characteristics of study participants (Chi-square test) in THIS2016/17, and THIS2022/23 (N=12,714)**

|  | **Ever tested for HIV** | | | |
| --- | --- | --- | --- | --- |
| **Variables** | **Adolescents(15-19yrs)** | | **Young women (20-25 yrs)** | |
|  | **2016/17**  **n=3602(%)** | **2022/23**  **n=3151(%)** | **2016/17**  **n=3046(%)** | **2022/23**  **n=2949(%)** |
| **Residence** |  |  |  |  |
| Rural | 39.3 | 39.5 | 86.9 | 91.5** |
| Urban | 40.9 | 40.8 | 85.2 | 87.5 |
| **Marital status** |  |  |  |  |
| Never in union | 28.7*** | 28.2*** | 74.3*** | 76.4*** |
| Currently in a union | 72.5 | 84.8 | 91.9 | 95.8 |
| Cohabiting | 76.9 | 83.3 | 92.5 | 95.8 |
| Formerly in union | 80.8 | 85.1 | 90.3 | 96.5 |
| **Occupation status** |  |  |  |  |
| Not employed | 38.3** | 37.5*** | 86.5 | 89.1 |
| Employed | 46.7 | 49.2 | 85.7 | 91.0 |
| **Education level** |  |  |  |  |
| No education | 45.3** | 57.8*** | 85.3** | 89.8*** |
| Primary education | 42.8 | 42.3 | 86.4 | 92.4 |
| Secondary/higher | 34.6 | 35.5 | 86.1 | 86.5 |
| **Zone** |  |  |  |  |
| Central | 39.2* | 32.5* | 86.4 | 86* |
| Lake | 37.4 | 39.9 | 86.7 | 89.9 |
| Northern | 32.5 | 35.1 | 81.9 | 86.7 |
| Eastern | 40.6 | 39.2 | 83.8 | 91.2 |
| South West Highland | 42.4 | 45.9 | 89.2 | 90.0 |
| Southern Highland | 54.5 | 53.7 | 89.2 | 94.8 |
| Southern | 39.6 | 35.9 | 88.9 | 91.2 |
| Western | 41.9 | 42.9 | 87.9 | 89.6 |
| **Household Wealth Index** |  |  |  |  |
| Poor | 39.7 | 40.6 | 85.5 | 92.1* |
| Middle | 47.0 | 40.9 | 89.6 | 89.0 |
| Rich | 38.8 | 38.8 | 86.2 | 88.0 |
| *Significant at P<0.05; **Significant at P<0.01; ***Significant at P<0.001 | | | | |

**S3 Table. (continued)**

|  | **Ever tested for HIV** | | | |
| --- | --- | --- | --- | --- |
| **Variables** | **Adolescents(15-19yrs)** | | **Young women(20-25yrs)** | |
|  | **2016/17 n=3602(%)** | **2022/23 n=3151(%)** | **2016/17**  **n=3046(%)** | **2022/23**  **n=2949(%)** |
| **Exposure to Radio/TV** |  |  |  |  |
| No | 39.7 | 41.2 | 85.5 | 92.1** |
| Yes | 39.9 | 39.6 | 86.6 | 88.6 |
| **Health insurance** |  |  |  |  |
| No | 39.4 | 40.8 | 85.7 | 90.5 |
| Yes | 43.7 | 36.6 | 90.3 | 87.3 |
| **Sexual debut** |  |  |  |  |
| <15 | 54.6 | 65.7 | 84.4* | 91.9* |
| 15+ | 63.5 | 69.8 | 89.8 | 93.3 |
| **Multiple sexual partners** |  |  |  |  |
| No partner | 60.5*** | 36.9*** | 85.5*** | 83.5*** |
| One | 63.1 | 66.1 | 90.8 | 93.3 |
| Two and above | 21.9 | 21.3 | 70.1 | 66.9 |
| **Condom use in the last sex** |  |  |  |  |
| No | 63.5*** | 73.8*** | 90.3*** | 93.6*** |
| Yes | 24.1 | 22.8 | 75.3 | 73.4 |
| **Had STI in the past 12months** |  |  |  |  |
| No | 62.6 | 33.2*** | 89.5 | 84.9*** |
| Yes | 55.5 | 82.3 | 87.7 | 97.7 |
| **HIV results from a biomarker test** |  |  |  |  |
| Negative | 39.6* | 39.6*** | 86.0 | 89.7 |
| Positive | 64.5 | 96.4 | 91.8 | 88.5 |
| *Significant at P<0.05; **Significant at P<0.01; ***Significant at P<0.001 | | | | |
