# Supplementary material for: Determinants of HIV testing uptake among adolescent girls and young women in mainland Tanzania: A stratified analysis of the 2016/17 and 2022/2023 national surveys
Source: PLoS One. 2026 Jul 8;21(7):e0343753. doi: 10.1371/journal.pone.0343753 (PMC13345389; doi:10.1371/journal.pone.0343753)
Supplement: S4 Table — (DOCX) [file pone.0343753.s004.docx]

**S4 Table. Multivariable Poisson regression on factors associated with HIV testing among AGYW in mainland Tanzania using data from THIS 2016/17 and 2022/23 (N=12,714)**

| **Variables** | **CPR (95%CI)** | **P-value** | **APR (95%CI)** | **p-value** |
| --- | --- | --- | --- | --- |
| **Age(years)** |  |  |  |  |
| 15-19 | 1 |  | 1 |  |
| 20-24 | 2.26(2.16-2.37) | <0.001 | 1.31(1.26-1.37) | <0.001 |
| **Residence** |  |  |  |  |
| Rural | 1 |  | 1 |  |
| Urban | 1.07(1.02-1.12) | 0.003 | 1.04(0.99-1.07) | 0.052 |
| **Zone** |  |  |  |  |
| Central | 1 |  | 1 |  |
| Lake | 0.99(0.93-1.05) | 0.996 | 0.96(0.88-1.06) | 0.872 |
| Northern | 0.99(0.85-1.15) | 0.817 | 1.00(0.87-1.17) | 0.660 |
| Eastern | 1.08(0.91-1.13) | 0.054 | 0.94(0.84-1.04) | 0.584 |
| SouthW highland | 0.99(0.88-1.17) | 0.952 | 0.99(0.88-1.11) | 0.992 |
| Southern highland | 1.01(0.88-1.17) | 0.876 | 1.01(0.89-1.14) | 0.763 |
| Southern | 1.13(0.99-1.29) | 0.222 | 1.09(0.97-1.23) | 0.717 |
| Western | 0.99(0.88-1.13) | 0.180 | 1.00(0.89-1.11) | 0.200 |
| **Marital status** |  |  |  |  |
| Never in union | 1 |  | 1 |  |
| Currently union | 2.1(2.00-2.27) | <0.001 | 1.29(1.20-1.28) | <0.001 |
| Cohabiting | 2.21(2.08-2.42) | <0.001 | 1.25(1.19-1.31) | <0.001 |
| Formerly in union | 2.35(2.14-2.53) | <0.001 | 1.20(1.20-1.34) | <0.001 |
| **Occupation status** |  |  |  |  |
| Not employed | 1 |  | 1 |  |
| Employed | 1.18(1.12-1.23) | <0.001 | 0.97(0.95-1.01) | 0.192 |
| **Education Level** |  |  |  |  |
| No education | 1 |  | 1 |  |
| Primary | 0.97(0.89-1.05) | 0.456 | 1.11(1.03-1.19) | 0.003 |
| Secondary/higher | 0.89(0.82-0.98) | 0.021 | 1.16(1.07-1.26) | <0.001 |
| **Wealth index** |  |  |  |  |
| Poor | 1 |  | 1 |  |
| Middle | 0.96(0.90-1.02) | 0.183 | 0.99(0.98-1.11) | 0.982 |
| Rich | 1.02(0.97-1.07) | 0.444 | 1.01(0.97-1.06) | 0.564 |
| 1-reference group; CPR-Crude Prevalence Ratio; APR-Adjusted Prevalence Ratio | | | | |

**S4 Table. (continued)**

| **Variables** | **CPR (95%CI)** | **p-value** | **APR (95%CI)** | **p-value** |
| --- | --- | --- | --- | --- |
| **Exposure to radio/TV** |  |  |  |  |
| No | 1 |  | 1 |  |
| Yes | 0.95(0.91-0.99) | 0.023 | 1.00(0.97-1.03) | 0.906 |
| **Had health insurance** |  |  |  |  |
| No | 1 |  | 1 |  |
| Yes | 1.07(1.01-1.14) | 0.029 | 0.95(0.88-1.020 | 0.867 |
| **Sexual debut** |  |  |  |  |
| <15 | 1 |  | 1 |  |
| 15+ | 2.05(1.86-2.26) | <0.001 | 1.03(0.94-1.14) | 0.813 |
| **Multiple sex partners** |  |  |  |  |
| No partner | 1 |  | 1 |  |
| One | 2.30(2.11-2.50) | <0.001 | 1.30(1.00-1.9) | 0.030 |
| Two or more | 0.91(0.81-1.01) |  | 0.85(0.77-1.05) | 0.107 |
| **Condom use** |  |  |  |  |
| No | 1 |  | 1 |  |
| Yes | 0.49(0.47-0.51) | <0.001 | 1.04(1.00-1.07) | 0.046 |
| **Had an STI in the last 12 months** |  |  |  |  |
| No | 1 |  | 1 |  |
| Yes | 1.69(1.64-1.76) | <0.001 | 1.22(1.18-1.26) | <0.001 |
| **HIV results from the biomarker test** |  |  |  |  |
| Negative | 1 |  | 1 |  |
| Positive | 1.37(1.27-1.49) | <0.001 | 1.06(0.91-1.22) | 0.238 |
| 1-reference group: CPR-Crude Prevalence Ratio; APR-Adjusted Prevalence Ratio | | | | |
