# Supplementary material for: Determinants of HIV testing uptake among adolescent girls and young women in mainland Tanzania: A stratified analysis of the 2016/17 and 2022/2023 national surveys
Source: PLoS One. 2026 Jul 8;21(7):e0343753. doi: 10.1371/journal.pone.0343753 (PMC13345389; doi:10.1371/journal.pone.0343753)
Supplement: S5 Table — (DOCX) [file pone.0343753.s005.docx]

**S5 Table. Multivariable modified Poisson regression on factors associated with HIV testing among AGYW in mainland Tanzania stratified by age using data from THIS 2016/17 and 2022/23 (N=12,714)**

|  | **15-19years** | | **20-24years** | |
| --- | --- | --- | --- | --- |
| **Variables** | **CPR(95%CI)** | **APR(95%CI)** | **CPR(95%CI)** | **APR(95%CI)** |
| **Residence** |  |  |  |  |
| Rural | 1 | 1 | 1 | 1 |
| Urban | 1.08(0.97-1.19) | 1.07(0.94-1.15) | 1.02(0.92-1.05) | 1.02(0.99-1.06) |
| **Zone** |  |  |  |  |
| Central | 1 | 1 | 1 | 1 |
| Lake | 1.04(0.88-1.22) | 0.95(0.83-1.10) | 1.017(0.96-1.08) | 1.00(0.95-1.07) |
| Northern | 0.99(0.77-1.27) | 1.03(0.81-1.18) | 0.97(0.87-1.09) | 1.01(0.91-1.17) |
| Eastern | 1.11(0.92-1.35) | 1.07(0.90-1.16) | 1.02(0.96-1.09) | 0.99(0.92-1.03) |
| SouthW Highland | 1.00(0.82-1.21) | 1.01(0.85-1.24) | 0.99(0.92-1.07) | 0.99(0.93-1.07) |
| Southern Highland | 1.09(0.87-1.37) | 1.01(0.83-1.14) | 0.99(0.92-1.07) | 1.00(0.93-1.08) |
| Southern | 1.13(0.92-1.39) | 0.97(0.85-1.33) | 1.06(0.98-1.14) | 1.02(0.95-1.28) |
| Western | 1.08(0.84-1.40) | 1.04(0.85-1.27) | 1.04(0.97-1.11) | 1.03(0.98-1.11) |
| **Marital** **status** |  |  |  |  |
| Never in union | 1 | 1 | 1 | 1 |
| Currently union | 2.65(2.4-2.90)*** | 1.52(1.38-1.68)*** | 1.21(1.16-1.28)*** | 1.12(1.07-1.17)*** |
| Cohabiting | 2.57(2.28-2.89)*** | 1.48(1.32-1.67)*** | 1.27(1.22-1.33)*** | 1.15(1.09-1.20)*** |
| Formerly in union | 2.86(2.54-3.23)*** | 1.61(1.41-1.85)*** | 1.24(1.18-1.31)*** | 1.13(1.06-1.24)*** |
| **Occupation status** |  |  |  |  |
| Not employed | 1 | 1 | 1 | 1 |
| Employed | 1.17(1.07-1.28)** | 0.95(0.87-1.13) | 0.98(0.95-1.02) | 0.98(0.96-1.02) |
| **Education Level** |  |  |  |  |
| No education | 1 | 1 | 1 | 1 |
| Primary | 0.85(0.72-1.01) | 1.08(0.94-1.25) | 1.15(1.06-1.26)** | 1.14(1.06-1.24)*** |
| Secondary/higher | 0.84(0.70-1.00) | 1.22(1.04-1.43)* | 1.11(1.01-1.22)* | 1.15(1.06-1.25)** |
| **Household wealth index** |  |  |  |  |
| Poor | 1 | 1 | 1 | 1 |
| Middle | 1.03(0.92-1.15) | 1.04(0.95-1.23) | 1.00(0.96-1.05) | 0.99(0.94-1.13) |
| Rich | 0.99(0.89-1.11) | 1.07(0.94-1.19) | 1.00(0.96-1.04) | 0.99(0.96-1.04 |
| *Significant at P<0.05; **Significant at P<0.01; ***Significant at P<0.001; 1-reference group: CPR-Crude Prevalence Ratio; APR-Adjusted Prevalence Ratio | | | | |

**S5 Table. (continued)**

| **Variables** | | | | **15-19years** | | | **20-24years** | |
| --- | --- | --- | --- | --- | --- | --- | --- | --- |
|  | | | | **CPR(95%CI)** | | **APR(95%CI)** | **CPR(95%CI)** | **APR(95%CI)** |
| **Exposure to TV/radio** | | | |  | |  |  |  |
| No | | | | 1 | |  | 1 |  |
| Yes | | | | 0.96(0.87-1.05) | |  | 0.99(0.96-1.04) |  |
| **Had health insurance** | | | |  | |  |  |  |
| No | | | | 1 | |  | 1 |  |
| Yes | | | | 0.98(0.86-1.12) | |  | 1.02(0.98-1.06) |  |
| **Sexual debut** | | | |  | |  |  |  |
| <15 | | 1 | | | | 1 | 1 | 1 |
| 15+ | | 2.43(2.17-2.71)*** | | | | 1.01(0.92-1.24) | 1.21(1.12-1.30)*** | 0.98(0.93-1.05) |
| **Multiple sex partners** | | | |  | |  |  |  |
| No partner | | | | 1 | | 1 | 1 | 1 |
| One | | 2.65(2.33-3.04)*** | | | | 1.30(0.88-1.91) | 1.25(1.15-1.35)*** | 1.65(0.89-1.04) |
| Two or more | | | | 0.96(0.82-1.14) | | 0.92(0.79-1.07) | 0.95(0.85-1.07) | 0.95(0.87-1.02) |
| **Condom use** | | | |  | |  |  |  |
| No | | | | 1 | | 1 | 1 | 1 |
| Yes | | | 0.42(0.37-0.47)*** | | | 1.07(0.95-1.18) | 0.88(0.83-.92)*** | 1.05(1.00-1.08)* |
| **Had an STI in the last 12months** | | | |  | |  |  |  |
| No | | | | 1 | | 1 | 1 | 1 |
| Yes | 2.32(2.12-2.54)*** | | | | 1.53(1.41-1.64)*** | | 1.19(1.16-1.24)*** | 1.12(1.09-1.15)*** |
| **HIV results from the biomarker test** | | | |  | |  |  |  |
| Negative | | | 1 | | | 1 | 1 | 1 |
| Positive | | | 1.98(1.66-2.36)*** | | | 1.04(0.82-1.30) | 1.00(0.92-1.09) | 1.04(0.94-1.12) |
| *Significant at P<0.05; **Significant at P<0.01; ***Significant at P<0.001; 1-reference group: CPR-Crude Prevalence Ratio; APR-Adjusted Prevalence Ratio | | | | | | | | |
