# Supplementary material for: Determinants of HIV testing uptake among adolescent girls and young women in mainland Tanzania: A stratified analysis of the 2016/17 and 2022/2023 national surveys
Source: PLoS One. 2026 Jul 8;21(7):e0343753. doi: 10.1371/journal.pone.0343753 (PMC13345389; doi:10.1371/journal.pone.0343753)
Supplement: S6 Table — (DOCX) [file pone.0343753.s006.docx]

| **Variables** | **CoR (95%CI)** | **P-value** | **AoR (95%CI)** | **p-value** |
| --- | --- | --- | --- | --- |
| **Age(years)** |  |  |  |  |
| 15-19 | 1 |  | 1 |  |
| 20-24 | 9.7(8.53-11.21) | <0.001 | 3.76(2.26-3.37) | <0.001 |
| **Residence** |  |  |  |  |
| Rural | 1 |  | 1 |  |
| Urban | 1.19(1.06-1.35) | 0.003 | 1.23(0.93-1.62) | 0139 |
| **Zone** |  |  |  |  |
| Central | 1 |  | 1 |  |
| Lake | 0.99(0.83-1.19) | 0.996 | 0.90(0.66-1.23) | 0.572 |
| Northern | 0.96(0.74-1.26) | 0.817 | 1.11(0.57-2.17) | 0.760 |
| Eastern | 1.22(0.99-1.50) | 0.054 | 1.10(0.74-1.66) | 0.533 |
| SouthW highland | 0.99(0.78-1.26) | 0.952 | 0.99(0.88-1.71) | 0.982 |
| Southern highland | 0.98(0.75-1.26) | 0.876 | 1.08(0.68-1.14) | 0.763 |
| Southern | 1.16(0.90-1.51) | 0.222 | 1.139(0.74-1.72) | 0.720 |
| Western | 1.18(0.92-1.52) | 0.180 | 1.22(0.81-1.85) | 0.340 |
| **Marital status** |  |  |  |  |
| Never in union | 1 |  | 1 |  |
| Currently union | 8.89(7.31-10.81) | <0.001 | 3.64(2.91-4.54) | <0.001 |
| Cohabiting | 10.56(8.25-13.51) | <0.001 | 3.89(2.85-5.31) | <0.001 |
| Formerly in union | 12.41(8.83-17.43) | <0.001 | 3.78(2.39-5.98) | <0.001 |
| **Occupation status** |  |  |  |  |
| Not employed | 1 |  | 1 |  |
| Employed | 1.51(1.35-1.69) | <0.001 | 1.22(0.99-1.51) | 0.060 |
| **Education Level** |  |  |  |  |
| No education | 1 |  | 1 |  |
| Primary | 0.91(0.72-1.16) | 0.456 | 1.87(1.38-2.55) | <0.001 |
| Secondary/higher | 0.76(0.59-0.98) | 0.021 | 2.45(1.67-3.59) | <0.001 |
| **Wealth index** |  |  |  |  |
| Poor | 1 |  | 1 |  |
| Middle | 0.91(0.79-1.04) | 0.183 | 1.09(0.85-1.39) | 0.510 |
| Rich | 1.50(1.37-1.65) | 0.444 | 1.323(1.00-1.71) | 0.045 |

**S6 Table: Multivariable logistic regression on factors associated with HIV testing**

**among AGYW in mainland Tanzania using data from THIS 2016/17 and 2022/23 (N=12,714)**

1-reference group; CoR-Crude odds Ratio; AoR-Adjusted odds Ratio

**S6 Table. (continued)**

| **Variables** | **COR (95%CI)** | **p-value** | **AOR (95%CI)** | **p-value** |
| --- | --- | --- | --- | --- |
| **Exposure to radio/TV** |  |  |  |  |
| No | 1 |  | 1 |  |
| Yes | 0.87(0.77-0.99) | 0.023 | 0.96(0.78-1.17) | 0.699 |
| **Had health insurance** |  |  |  |  |
| No | 1 |  | 1 |  |
| Yes | 1.19(1.01-1.14) | 0.029 | 0.93(0.70-1.24) | 0.634 |
| **Sexual debut** |  |  |  |  |
| <15 | 1 |  | 1 |  |
| 15+ | 6.97(6.04-8.05) | <0.001 | 1.01(0.74-1.36) | 0.813 |
| **Multiple sex partners** |  |  |  |  |
| No partner | 1 |  | 1 |  |
| One | 7.14(6.14-8.31) | <0.001 | 2.10(1.52-3.30) | 0.001 |
| Two or more | 0.86(0.73-1.01) | 0.081 | 0.71(0.49-1.05) | 0.065 |
| **Condom use** |  |  |  |  |
| No | 1 |  | 1 |  |
| Yes | 0.15(0.13-0.18) | <0.001 | 1.58(1.25-2.00) | <0.001 |
| **Had an STI in the last 12 months** |  |  |  |  |
| No | 1 |  | 1 |  |
| Yes | 9.32(7.23-12.02) | <0.001 | 2.00(1.51-2.65) | <0.001 |
| **HIV results from the biomarker test** |  |  |  |  |
| Negative | 1 |  | 1 |  |
| Positive | 3.13(2.01-4.88) | <0.001 | 1.32(0.75-2.34) | 0.338 |

1-reference group; CoR-Crude odds Ratio; AoR-Adjusted odds Ratio
